# Effect of Si and C additions on the reaction mechanism and mechanical properties of FeCrNiCu high entropy alloy

**DOI:** 10.1038/s41598-019-52809-y

**Published:** 2019-11-08

**Authors:** Hao Wu, Sirui Huang, Huan Qiu, Heguo Zhu, Zonghan Xie

**Affiliations:** 1 College of Materials Science and Engineering, Nanjing University of Science and Technology, Nanjing, 210094 P.R. China; 2 School of Mechanical Engineering, University of Adelaide, SA 5005 Adelaide, Australia; 3 School of Engineering, Edith Cowan University, WA 6027 Joondalup, Australia

**Keywords:** Mechanical engineering, Composites

## Abstract

FeCrNiCu based high entropy alloy matrix composites were fabricated with addition of Si and C by vacuum electromagnetic induction melting. The primary goal of this research was to analyze the reaction mechanism, microstructure, mechanical properties at room temperature and strengthening mechanism of the composites with addition of Si and C. The reaction mechanism of powders containing (Si, Ni and C) was analyzed, only one reaction occurred (i.e., Si + C → SiC) and its activation energy is 1302.8 kJ/mol. The new composites consist of a face centered cubic (FCC) structured matrix reinforced by submicron sized SiC particles. The addition of Si and C enhances the hardness from 351.4 HV to 626.4 HV and the tensile strength from 565.5 MPa to 846.0 MPa, accompanied by a slight decrease in the plasticity. The main strengthening mechanisms of SiC/FeCrNiCu composites were discussed based on dislocation strengthening, load bearing effect, Orowan mechanism and solid solution hardening, whose contributions to the tensile strength increase are 58.6%, 6.3%, 14.3% and 20.8%, respectively.

## Introduction

Traditionally, a small amount of alloying elements are often added into a base metal to improve the mechanical properties of the material. The resulting alloy is typically classified by the base element, such as ferrous or aluminium alloy. Different from traditional alloys, high entropy alloys (HEAs) consist of multiple principal elements^1,2^. Instead of forming intermetallics, HEAs are single solid solutions made of face centered cubic (FCC)^3,4^, body centered cubic (BCC)^5,6^ and hexagonal close-packed (HCP)^7^ or the mixture of them^8,9^, manifest of the high mixing entropy and sluggish interdiffusion characteristic^1,2,10^. HEAs have shown advantages of high thermodynamic stability^11^, combination of high strength and ductility^12–16^, good abrasive resistance^17,18^ and corrosion resistance^19^, suitable for applications involving harsh environment^20–22^.

Ceramic particles have been used as reinforcement in high entropy alloy matrix composites, which have distinct properties with regard to the matrix. For example, AlZnMgCuZr lightweight high entropy alloy based composites were prepared by incorporating 10% of TiB_2_^23^; the compressive strength, hardness and elastic modulus of the composites were greatly enhanced due to the grain refinement. Lukasz *et al*.^24^ prepared CoCrFeMnNi high entropy alloy matrix composites reinforced with Al_2_O_3_ nano-particles. Compared with CoCrFeMnNi matrix, the hardness of the composites increases from 418 HV to 515 HV, while the yield strength was enhanced by 35.6%.

*In-situ* composite, in which one or more enhancement phases are produced by chemical reactions, exhibit some unique advantages over *ex-situ* composites^25^. For example, the reinforcement is uniformly distributed in the matrix and the reinforcement and the matrix is relatively stable with clean interface. So far, there have been a small number of researches on the production of high entropy alloy matrix composites reinforced with *ex-situ* SiC particles^26^, but little report about composites reinforced with *in-situ* SiC particles. In this paper, HEA composites containing FeCrNiCu matrix and *in-situ* SiC particles were prepared by vacuum electromagnetic induction melting. The microstructure and mechanical properties of the composites were characterized and the strengthening mechanisms analyze.

## Results and Discussion

### DSC analysis

**Figure 1:**
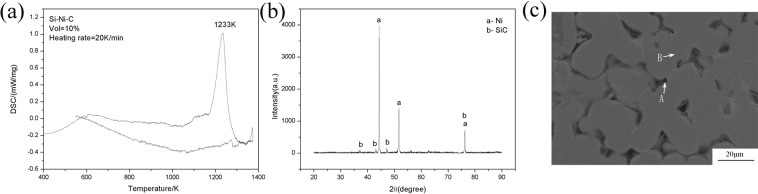
(**a**) DSC curve of Si-Ni-C system; (**b**) XRD diffraction pattern of Si-Ni-C system;(**c**) SEM image of Si-Ni-C system.

The DSC curve of the Si- C system (Fig. 1a) has a peak at 1233 K, signifying a reaction took place during the heating stage. The XRD diffraction pattern of the specimen prepared from heating up to 1300 K (Fig. 1b) shows the diffraction peaks of Ni and SiC^27,28^. The SEM image (Fig. 1c) suggests the possible existence of Ni and SiC. The EDS analysis indicates that the gray region is made of Ni, while the dark region is composed of Si and C, which suggests the formation of SiC (Table 1) via:

1 $$S{\rm{i}}+C\to SiC\,\,\Delta {G}^{0}=-123470+37{\rm{.6T}}\,{\rm{J}}/{\rm{mol}}$$

**Table 1:** Chemical compositions of different phases measured by SEM/EDS (areas marked in Fig. 1c).

Figure	Area	Element	(at.%)
		Ni	Si
Fig. 1c	A	28.53	71.47
	B	97.41	2.59

**Figure 2:**
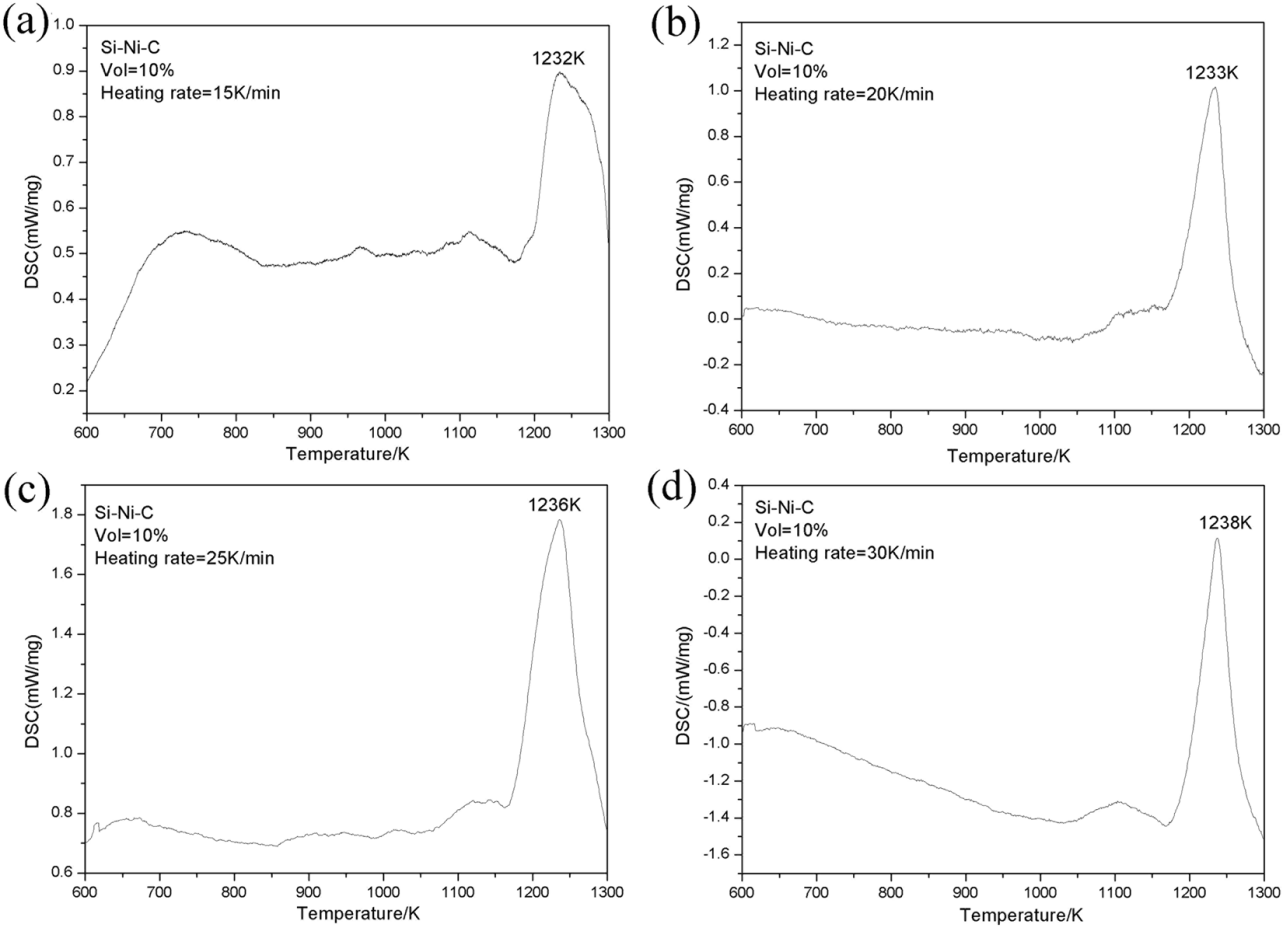
DSC curves obtained at four different heating rates, (**a**) 15 K /min; (**b**) 20 K /min; (**c**) 25 K /min; (**d**) 30 K /min.

**Figure 3:**
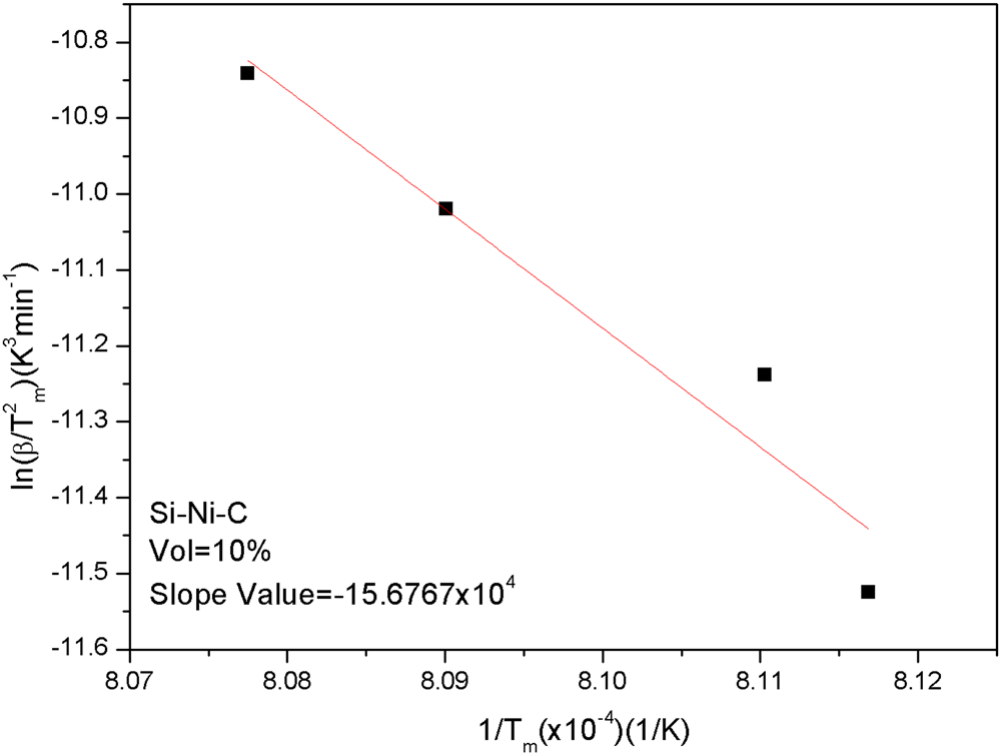
Plots of the ln(*β*/*T*^2^_*m*_) − 1/*T*_*m*_ for the reactions of Eq. (1).

The SC10 was taken as an example to determine the apparent activation energy of chemical reactions during materials synthesis. Figure 2 shows the DSC curve acquired at the heating rate of 15 K/min, 20 K/min, 25 K/min and 30 K/min, respectively. With the increase of the heating rate, the reaction peak became sharper and the reaction temperature was moving upward. According to Kissinger’s equation^29^, the activation energy, *E*, of the reaction can be defined as:

2 $$\frac{d(\mathrm{ln}\,\frac{\beta }{{T}_{m}^{2}})}{d(\frac{1}{{T}_{m}})}=-\frac{E}{R}$$

where *T*_*m*_ is the peak temperature of the reaction, *β* is the heating rate, and *R* is ideal gas constant (i.e., 8.31 J/mol). The peak temperature of the reactions at different heating rates (i.e. 15 K/min, 20 K/min, 25 K/min, and 30 K/min) can be obtained from Fig. 2. The relationship between ln(*β*/*T*^2^_*m*_) − 1/Tm can be plotted and fitted linearly through discrete points and the slope value is determined, which is −15.6767 × 10^4^ (Fig. 3). The activation energy was calculated and found to be 1302.8 kJ/mol. The result shows that the formation of SiC requires a large amount of energy input and is thus difficult to form (at about 1233 K).

### Microstructural characteristics

**Figure 4:**
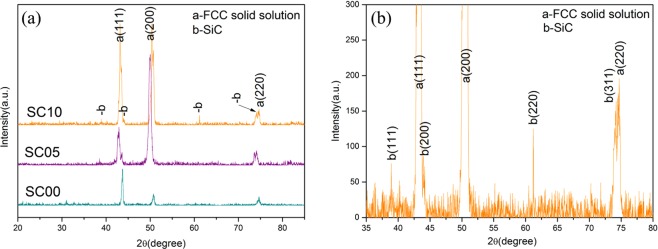
(**a**) XRD diffraction patterns of FeCrNiCu high entropy alloy matrix composites with different volumes of reinforcement agents; (**b**) Enlarged patterns of the HEA with 10% vol.SiC.

Figure 4a shows that XRD pattern of FeCrNiCu high entropy alloy matrix composites prepared with different additions of Si and C. The matrix is found to be FCC single phase solid solution. With the addition of Si and C, the diffraction peaks of SiC start to appear and becomes intensified with new SiC peak appearing at 2θ ≈ 61°, when the content of Si and C continues to increase. Compared with the base alloy, the FCC diffraction peaks of the composites is shifted to the left, presumably due to the lattice distortion caused by incorporation of Si and C. Figure 4b presents the enlarged diffraction pattern of the SC10, revealing the presence of the SiC particles with a FCC structure.

**Figure 5:**
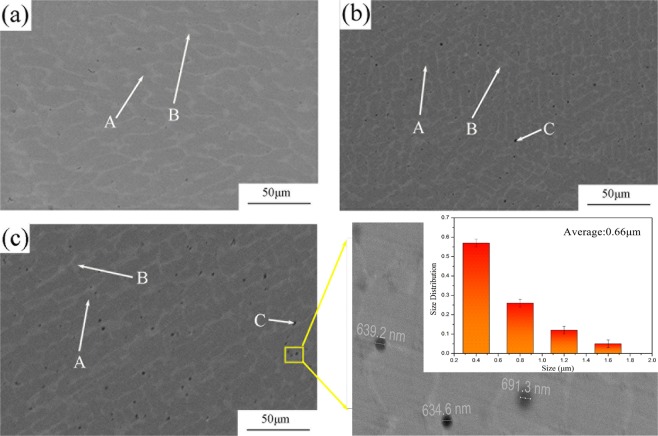
(**a**) SEM micrograph of FeCrNiCu high entropy alloy; (**b**) SEM micrograph of 5. vol% SiC/FeCrNiCu high entropy alloy matrix composite; (**c**) SEM micrograph of 10. vol% SiC/FeCrNiCu high entropy alloy matrix composite.

It can be seen from Fig. 5 that compared with the base alloy, the SiC reinforced composites exhibit drastic changes. In Fig. 5a, FeCrNiCu HEA has a homogeneous microstructure. With the addition of Si and C, SiC particles appear and are distributed uniformly in the matrix (Fig. 5b,c). The size of SiC particles was measured and found to be about 0.66 μm. The reinforcement content was analyzed using Image J software. In SC05 and SC10, the content of SiC is 3.5% and 6.4%, respectively.

Table 2 shows the matrix composition of the samples. Cu-rich and Cu-poor regions can be identified. Formation of these distinct regions may be explained by the ∆H_mix_ value between Cu and other atoms. The ∆H_mix_ values of Cu-Cr,Cu-Fe and Cu-Ni are 12 kJ/mol, 13 kJ/mol and 4 kJ/mol, respectively^30^. The ∆H_mix_ value of Cu-Ni represents the lowest. With the formation of SiC particles, large congregations of Cu were becoming more apparent.

**Table 2:** Chemical compositions of different phases in the FeCrNiCu HEA and its composites by SEM/EDS (areas marked in Fig. 5).

Specimen	Area	Element	(at.%)			
		Fe	Cr	Ni	Cu	Si
SC00	A	30.90	29.95	27.17	11.98	—
	B	10.77	11.98	14.29	62.96	—
SC05	A	28.13	27.87	24.33	18.70	0.97
	B	9.48	7.52	19.14	62.59	1.26
	C	17.16	16.41	15.97	32.78	17.68
SC10	A	33.34	26.02	27.93	11.64	1.06
	B	8.19	7.08	12.41	70.75	1.57
	C	14.25	14.51	13.08	26.30	31.85

**Figure 6:**
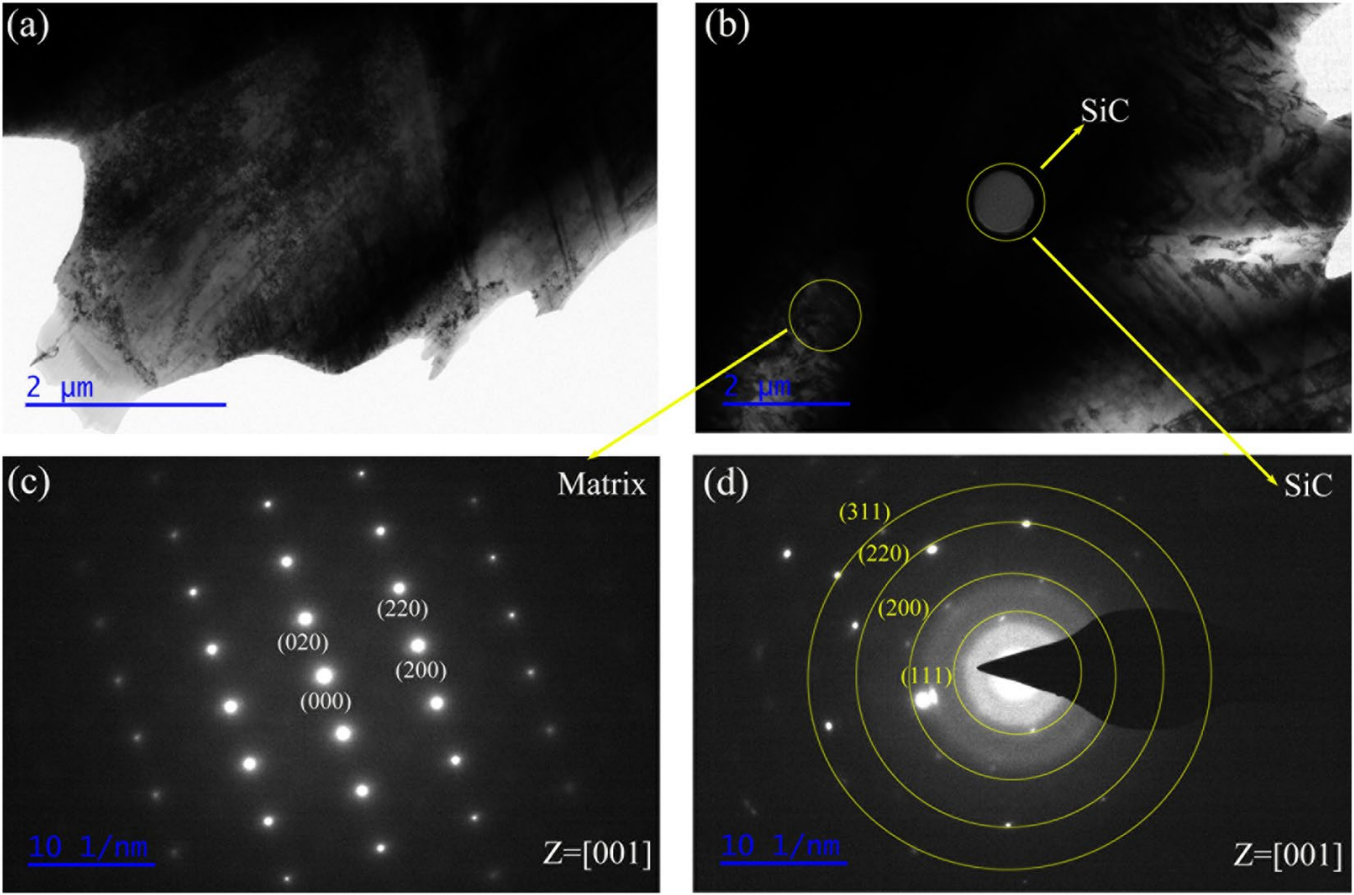
(**a**) TEM micrograph of FeCrNiCu high entropy alloy; (**b**) TEM micrograph of 10. vol% SiC/FeCrNiCu high entropy alloy matrix composite; (**c**) the SAED of the FeCrNiCu matrix; (**d**) the SAED of the SiC particles.

Figure 6 shows the detailed microstructure of SC00 alloy and SC10 composite. SiC particles have a circular shape. The selected area diffraction (SAED) pattern acquired from the matrix also confirm the FCC structure in the matrix. The diffraction pattern of the SiC particles is presented, along with the corresponding crystal plane exponents and lattice constants.

### Mechanical properties

**Figure 7:**
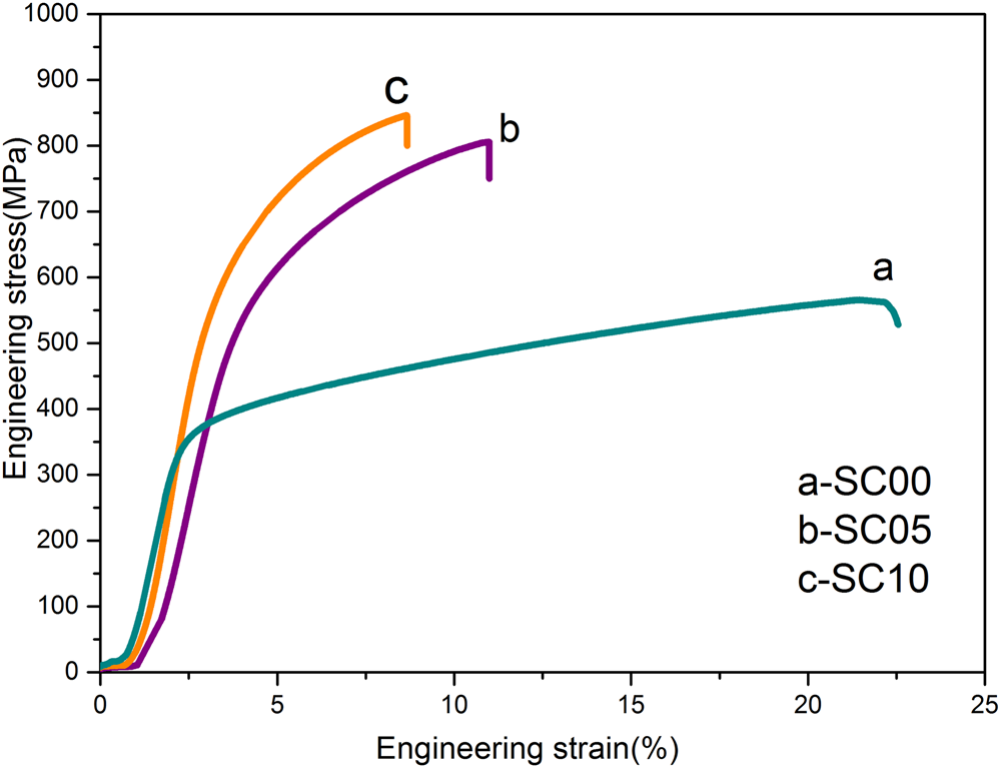
Engineering stress-strain curves of HEAs with different volume fractions of reinforcements.

The engineering stress-strain curves of the FeCrNiCu matrix composites with different volume fractions of SiC at room temperature are displayed in Fig. 7. For comparison purposes, Table 3 lists the mechanical properties of these composites. The FeCrNiCu HEA shows high ductility with the plastic strain reaching 21.5%, while the tensile strength and hardness of the base alloy are only 565.5 MPa and 351.4 HV, respectively. In comparison, the FeCrNiCu composites show a marked enhancement in hardness up to 626.4 HV. The tensile strength also increases from 565.5 MPa to 846.0 MPa (representing a 49.6% increase), accompanied by a decrease in ductility (i.e., 8.7%).

**Table 3:** Mechanical properties of the FeCrNiCu high entropy alloy with different volume fractions of SiC.

Sample	*σ*_*E*_/MPa	*ε*_*E*_/%	HV
SC00	565.5	21.5	351.4
SC05	805.6	11.0	601.6
SC10	846.0	8.7	626.4

### Fracture morphology

**Figure 8:**
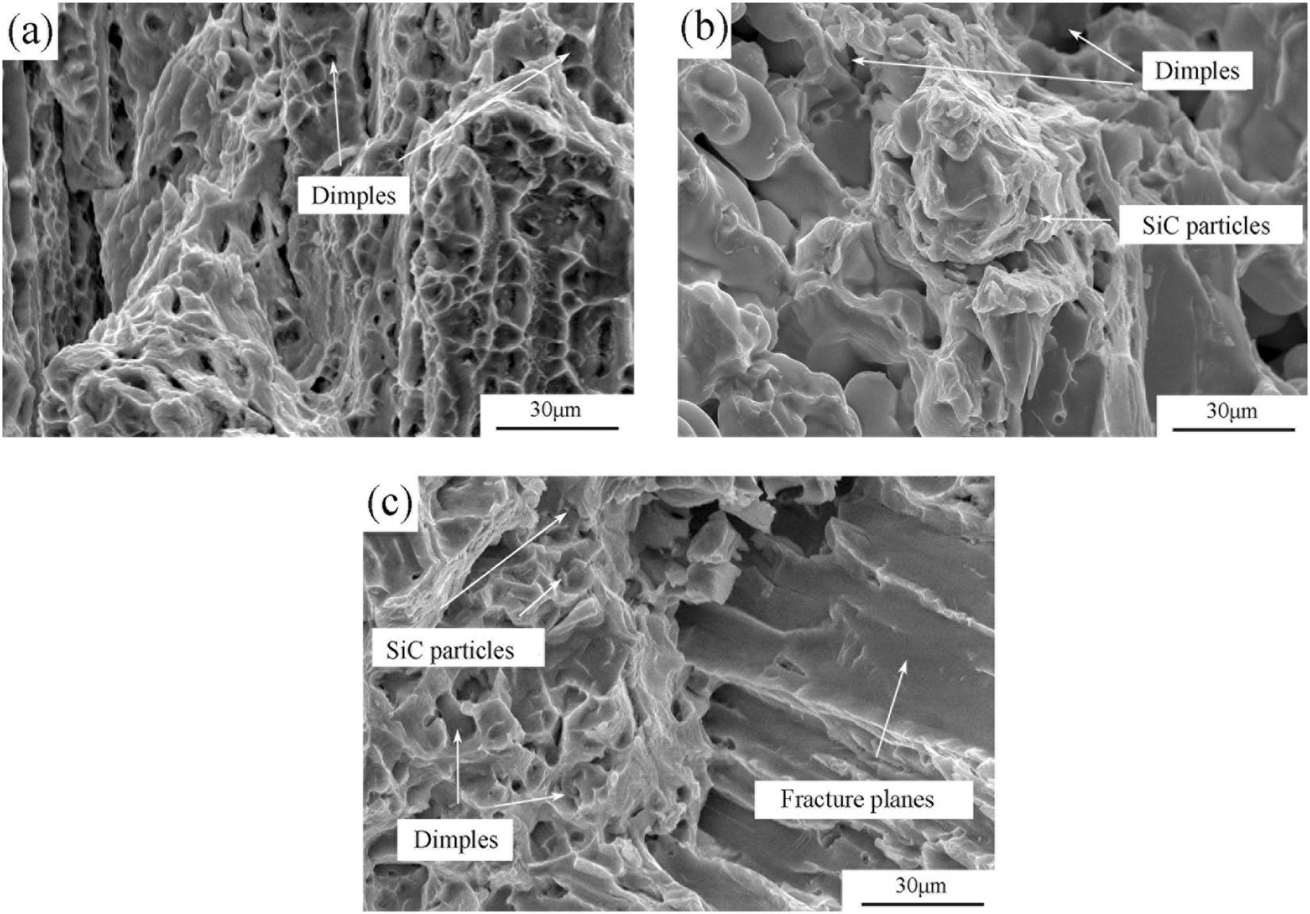
Fracture morphology of HEAs with different volume fractions of reinforcements, (**a**) matrix; (**b**) 5%; (**c**) 10%.

The tensile fracture surfaces of base alloy exhibit a large amount of small dimples (Fig. 8a), revealing ductile fracture pattern. Intact SiC particles are observed within the dimples (Fig. 8b), suggesting a strong interface bonding between the SiC particles and high entropy alloy matrix. When the volume fraction of SiC is 10%, the fracture morphology of the composite materials is shown in Fig. 8c. Small dimples and brittle fracture planes co-existed, revealing that the fracture was controlled by a mix of ductile and brittle processes. This resulted in a decline in material ductility. The above results are consistent with Table 3.

### Strengthening mechanisms

**Figure 9:**
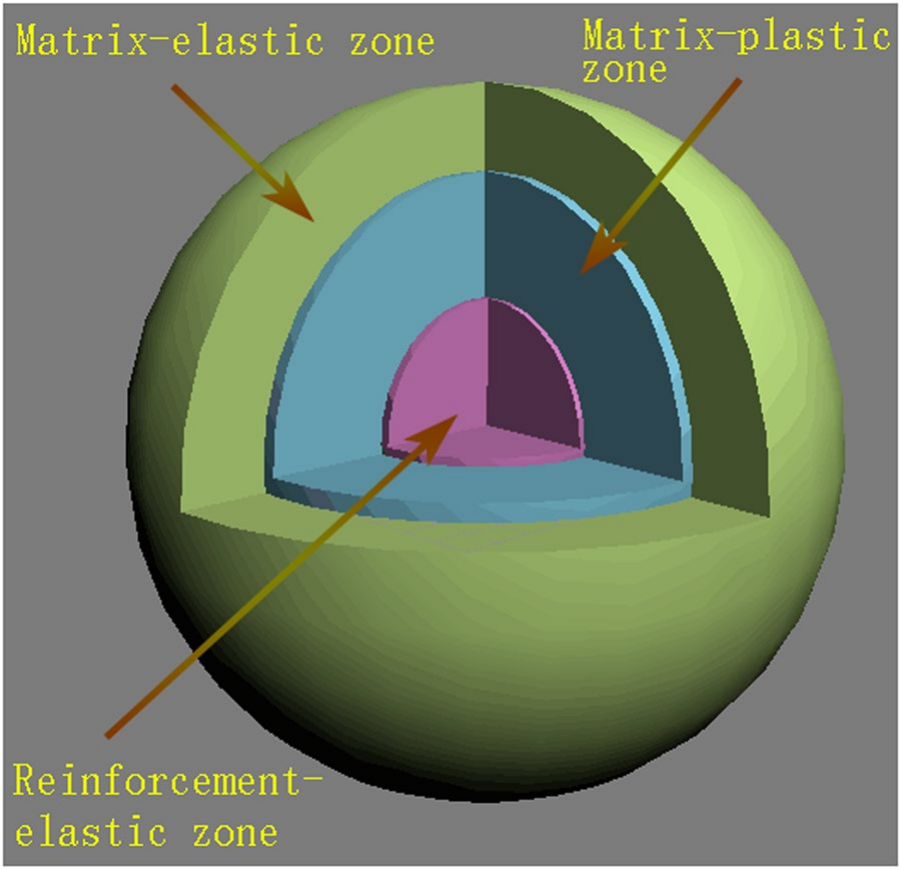
Sphere approximation of particles reinforced matrix composites.

The design of metal matrix composite is governed by the principle that the applied load can be transmitted to the reinforcement agents which are the main undertaker of the load^31^. The high density dislocations formed in the matrix material during synthesis also play an important role in strengthening the metal matrix composites^32^. Ramakrishnan *et al*. combined the role of the load-bearing effect and the dislocation strengthening mechanism in understanding the origin of a composite’s strength^33^. A composite model consists of three distinctive components; that is, elastic reinforcements, surrounding matrix-plastic zones and peripheral elastic areas, as exhibited in Fig. 9. Assuming that the plastic zone is governed by the ideal plastic state and the volume is constant. The rheological stress can be described by Mises’s effective stress. The external boundary conditions are regulated by free radial stress. The radial stress and the shear strain are continuous in the matrix-plastic and matrix-elastic zones as the same as at the particle-matrix interface. The Mises’s effective stress is continuous across the interface between matrix-plastic zone and matrix-elastic zone. The tensile strength of composites can be expressed as:

3 $${\sigma }_{cy}={\sigma }_{y}\times (1+{f}_{l})(1+{f}_{d})$$

where *σ*_*cy*_ and *σ*_*y*_ are the tensile strengths of the SC10 composite and SC00 alloy, respectively; *f*_*l*_ and *f*_*d*_ are the correction factor of load-bearing effect and the dislocation strength, respectively. The *f*_*l*_ and *f*_*d*_ can be defined as follows:

4 $${f}_{l}=0.5{V}_{P}$$

5 $${f}_{d}=\frac{k{G}_{m}b{\rho }^{\frac{1}{2}}}{{\sigma }_{y}}$$

where *V*_*P*_ is the volume fraction of reinforced particles (~6.4%), *k* is a coefficient (~1.6), *G*_*m*_ is the shear modulus of the matrix (~102.4 GPa), *b* is the Burger vector (~0.286 nm^23^) and the dislocated density *ρ* can be calculated by the equation below:

6 $$\rho =\frac{9.6\Delta \alpha \Delta T{V}_{P}}{bd}$$

where *∆α* is the value of difference between the thermal expansion coefficients for the SiC reinforcement particles (~4.3 × 10^−6^ K^−1^ ^34^) and the matrix (~approximately 0^35^), ∆*T* is the value of difference between room temperature (~293 K) and processing temperature (~1233 K), *V*_*P*_ is the volume fraction of the reinforcements and *d* is the average size of SiC particles (~ 0.66 μm).

Apart from the load-bearing effect and the dislocation strengthening, Orowan strengthening also contributes to the tensile strength of the composites. Orowan strengthening is generated by the interactions between dispersed reinforcements and dislocations. Accordingly, the tensile strength of the composites enhanced by *in-situ* SiC particles can be written as follows^36^:

7 $$\Delta {\sigma }_{O}=\frac{0.13{G}_{m}b}{\lambda }\,\mathrm{ln}(\frac{d}{2b})$$

where *G*_*m*_ is the shear modulus of the matrix, *b* is the Burger vector, *d* is the average size of SiC particles, and *λ* is the distance between the particles, which can be described as follows:

8 $$\lambda =d({(\frac{1}{2{V}_{P}})}^{\frac{1}{3}}-1)$$

where *V*_*P*_ is the volume fraction of *in-situ* SiC particles.

The solid solution strengthening is also one of important strengthening mechanisms. Solid solution strengthening is mainly realized by the uniform distribution of constituent atoms. When atoms are dissolved in the matrix to form solid solution, lattice distortion would occur in the matrix. The stress field caused by lattice distortion interacts with the stress field around the dislocations, which immobilizes the dislocations. As a result, the shear stress required for dislocation slip is increased in order to overcome the pinning effect. The rise of the tensile strength *Δσ*_*solute*_ due to the solid solution strengthening can be expressed as^37^:

9 $$\Delta {\sigma }_{solute}=MG(\frac{{{\varepsilon }_{{\rm{SS}}}}^{\frac{3}{2}}}{700}){c}^{\frac{1}{2}}$$

where *M* is the Taylor constant (~3.06 for FCC metals^38^), *G* is the shear modulus of the matrix, *c* is the molar mass concentration of the solutes (~2.18 at. %) and ε_SS_ is a coefficient associated with the fractional change in lattice coefficient per unit concentration of solute atom, which is in close connection with the atomic size of solutes^39^. Count of ε_SS_^3/2^/700 for Si not reachable on account of the short of proper data. Because of the analogous atomic sizes, the date of ε_SS_^3/2^/700 is similar to 1.3 × 10^−3^ ^39^. The ultimate tensile strength of the SC10 composite can be shown as:

10 $${\sigma }_{cy}={\sigma }_{y}\times (1+{f}_{l})(1+{f}_{d})+\Delta {\sigma }_{O}+\Delta {\sigma }_{solute}$$

The ideal or theoretical tensile strength can be calculated as:

11 $${\sigma }_{cy}=565.5\times (1+0.032)\times (1+0.30)+41.30+60.14=860.11MPa$$

The highest contribution to the tensile strength thus came from the dislocation strengthening, which accounts for 58.6%. Three other contributions; i.e., the load bearing effect, Orowan strengthening and solid solution strengthening, account for 6.3%, 14.3% and 20.8%, respectively. The theoretical tensile strength of the composites (860.11 MPa) are in good agreement with experimental data (846.0 MPa). The small difference may be due to a small deviation in the size of SiC particles.

## Conclusions

The *in-situ* composites containing of FeCrNiCu high entropy alloy matrix and SiC particles were designed and prepared by vacuum electromagnetic induction melting. The reaction process of the Si-Ni-C system consists of one step; i.e., Si reacting with C to form SiC particles as reinforcement phase. The activation energy of the reaction was found to be 1302.8 kJ/mol. The matrix of the as-sintered composites is mainly composed of Cu-rich phase and Cu-poor phase. The mechanical properties of the composites are significantly improved by the presence of SiC reinforcement phase. The hardness of SC10 is 78.3% higher than that of the matrix. The tensile strength of the composite is 846.0 MPa, which is 49.6% greater than that of the matrix. The multiple strengthening mechanisms were identified; namely, dislocation strengthening, load bearing effect, Orowan mechanism and solid solution strengthening. Among them the major contribution is from dislocation strengthening, which raised the tensile strength of the matrix by 58.6%.

## Methods

Silicon powders (15–25 µm in radius), carbon powders (0.5–3 µm in radius), nickel powders (15–25 µm in radius), iron particles (0.5–1 mm in radius and 5 mm in length), copper particles (0.5–1 mm in radius and 5 mm in length) and chromium particle (0.5–1 mm in radius and 5 mm in length) were used as raw materials. Each of them has a purity of 99.9%. The volume fraction of Si and C in the high entropy alloy matrix composites was designed as 5% and 10%, respectively. Hence the resulting materials are designated as SC00 (Si and C-free), SC05 and SC10 composites, respectively. To begin with, the powders of Si, Ni and C were blended and ball-milled in a vacuum stainless steel jar under the speed of 250 rounds/min for 8 hours. Then the mixed powders were forged into small blocks under the pressure of 150 MPa at room temperature. The small blocks (containing Si, Ni and C) was further processed by high temperature sintering at 1373 K for 2 hours. The HEA composites were generated by vacuum electromagnetic induction melting. The three elemental particles (Fe, Cr and Cu) and prefabricated blocks were placed in a ceramic crucible inside an oven prior to induction melting. The vacuum level of the oven was reduced to 5 × 10^−3^ Pa by the mechanical and molecular pumps. The current was set to be 550 A, which was reduced to 300 A for electromagnetic stirring after the materials were fused. The molten metal was poured into a copper crucible and cooled to room temperature inside the furnace.

The green compact samples made from Si, Ni and C powders (each about 5–10 mg) were placed in a thermal analyzer (DSC, STA449C). The heating temperature was increased from the room temperature (293 K) to 1373 K at four different heating rates (i.e., 15 K/min, 20 K/min, 25 K/min and 30 K/min) before cooling down to 473 K at the rate of 30 K/min. Using four different types of DSC curve generated from these measurements, the apparent activation energy of the reaction system (Si-Ni-C) was calculated. The crystal structure of sintered materials including HEA composites was characterized by means of X-ray diffraction (XRD, Bruker-AXS D8 Advance) through the CuKα filtered ray scanning at a scanning speed of 4°/min. To observe the microstructure and tensile fracture morphology, a scanning electron microscope (SEM, Quant 250FEG) was used. The compositions of the HEA matrix composites were investigated by the energy-dispersive spectrometry (EDS, Quant 250FEG). ImageJ software was utilized to estimate the volume fractions of SiC particles for the two composites using the scanning electron microscope images obtained at 3000× magnification; at least three SEM images were examined for each type of sample. The transmission electron microscopy (TEM,TECNAI G2 20 LaB6) was also used to further characterize the crystal structures and microstructures of the samples. The tensile tests were performed using a universal testing machine (UTM/CMT 5000) at a strain speed of 0.5 mm/min at room temperature. The tensile samples (length ~10.6 mm; width ~2.4 mm; and thickness ~1.2 mm) were wire cut from the ingots and then mechanically polished with emery paper to remove the surface defects. Vickers hardness tests were conducted using a hardness tester (HVS-1000). The maximum load was 5 N and the measurements were carried out in five different regions for each sample to ensure accuracy and confidence.
